# The Influence of Adhesive Compounds Biochemical Modification on the Mechanical Properties of Adhesive Joints

**DOI:** 10.3390/polym10040344

**Published:** 2018-03-21

**Authors:** Anna Rudawska, Izabela Haniecka, Magdalena Jaszek, Dawid Stefaniuk

**Affiliations:** 1 Faculty of Mechanical Engineering, Lublin University of Technology, 20-618 Lublin, Poland; i.haniecka@wp.pl; 2 Department of Biochemistry, Maria Sklodowska-Curie University, 20-033 Lublin, Poland; magdalena.jaszek@poczta.umcs.lublin.pl (M.J.); dawid.stefaniuk@poczta.umcs.lublin.pl (D.S.)

**Keywords:** epoxy adhesive, biochemical modification, adhesive joints, mechanical properties

## Abstract

The main purpose of this paper was to determine the effect of biochemical modification of epoxy adhesive compounds on the mechanical properties of hot-dip galvanized steel sheet DX51+Z275 adhesive joints. The epoxy adhesives (resin and curing agent) were biochemically modified by lyophilized fungal metabolites (in the form of lyophilized fungal fractions or materials preparation containing low molecular weight secondary metabolites of lignocellulose-degrading white rot fungi (WRF) *Pycnoporus sanguineus* (L.) *Murrill* and prepared by two methods). The epoxy adhesives (epoxy resin Epidian 53 and poliaminoamide curing agent PAC) were biochemical modified by lyophilized fungal metabolites and prepared by two methods. In the first method (Method I), the epoxy resin and the curing agent were mixed with the fungal material in the desired concentration. In the second method (Method II), the resin was mixed with mortar-grounded lyophilized post-culture liquid of the desired concentration and after following thorough mixing, a suitable amount of the poliaminoamide curing agent was added. The single-lap adhesive joints were prepared by modified epoxy adhesive compounds and were cured in various climatic factors. The specimens of adhesive joints were cured at single stage at the same temperature and humidity as during adhesive bonding (Variant A and Variant B). At the second stage, Method I adhesive joints were seasoned for two months at the temperature of 50 °C and 50% humidity in a climate test chamber (Variant C). The shear strength tests of the single-lap adhesive joints were performed using a Zwick/Roell Z150 testing machine in accordance with the DIN EN 1465 standard. The analysis of results revealed that the addition of the biological modifier can lead to reduced adhesive joint strength in ambient conditions, yet at elevated temperature and the higher humidity it results in a significant increase in adhesive joint strength.

## 1. Introduction

The dynamic development of adhesion bonding techniques started in the twentieth century and continues to this day. This is due to the possibilities offered by this process compared to other traditional joining methods (soldering, pressure welding, and welding). Given reduced costs and labor consumption, as well as a wide range of available binding materials for the adhesive bonding of various substrates depending on the needs, this method is one of the most promising joining technologies. Besides their primary role of bonding material, adhesive joints can also play supporting roles such as sealing, clamping, and securing. Due to these properties, the adhesive bonding technology has been widely used in a wide range of industry sectors such as aviation, astronautics, automotive, construction, electronics, and electrical engineering industries [1,2,3,4,5].

Nowadays many research centers in the world conduct research on the adhesive bonding technology which combines information from various areas of science. The research involves investigation of problems which have a significant impact primarily on adhesive joint strength which defines load-carrying capacity. Adhesive joint strength depends on factors such as physical and chemical phenomena (wettability, adhesion, and cohesion [1]), technological factors (surface treatment and metrological aspects of surface characteristics e.g., surface roughness, surface texture [2,6,7,8,9], and constructional factors (dimensions, loading, and type of joints [3,10]) affecting correct design of adhesive joints, and operating conditions affecting adhesive joints during operation [10,11].

Epoxy adhesives are the most popular kind of commercial adhesives [12,13,14,15]. This is due to their excellent mechanical properties (high resistance to load and tensile strength) and chemical properties (high resistance to chemicals and temperature, as well as good adhesion to aluminum, steel, and many other plastics). Given a wide selection of both resins and curing agents for preparing adhesives, it is possible to obtain a product that is the most suitable for given operating conditions [15,16,17,18]. Therefore, epoxy adhesives have been applied in many sectors of industry [19], from aircraft construction [1,3], through to the production of epoxy coatings [20,21,22].

Epoxy resins can be modified in an almost infinite number of ways using other polymers or resins [23,24,25,26,27,28,29] and also by the addition of different modifiers such as mineral fillers and nanofillers [26,27,28] and lignin fillers [30,31,32,33,34]. Lignin has been used in epoxy resin, and many different formulation approaches have been investigated [35,36]. Lots of the natural substances (polysaccharides, proteins, nucleic acids, phospholipids, or surfactants) can probably be an interesting source of adhesive modifiers [37].

Wood-decaying fungi, particularly white rot fungi, have attracted the interest of several researchers due to their remarkably effective biodegradation system [38,39,40,41]. Given their effective production of diverse secondary metabolites (e.g., enzymes such as laccase), these organisms have been long used in different areas of biotechnology [32]. Laccase is also used in the research on modification of the adhesive properties of plant biopolymers such as lignin [32,42] which can be found in resins or adhesives for the adhesive bonding of wood [29,33,34,35,43]. Given the metabolic biodiversity of wood-decaying fungi, the use of wood-decaying fungi has good prospects in the research on adhesive compound modification.

The composition and the properties of *Pycnoporus sanquineus*, a low molecular weight subfraction used as a modifying agent in the present work, are precisely described in [44]. Besides the unique qualitative composition of the fungal preparations used in this work, they also seem noteworthy especially in the context of adhesive mixtures modification as well as due to their very high antioxidant and antibacterial potential.

Based on the results of preliminary tests, it can be supposed that the use of metabolites derived from fungal cultures as modifiers for epoxy compounds could have a positive effect on not only the strength of adhesive joints exposed to various climatic conditions but also ageing and degradation processes of adhesive compounds. In a previous work [44], the authors investigated the mechanical properties of modified epoxy adhesive compounds by biological material, i.e., the lyophilized fungal preparation: lignin cellulose-degrading *P. sanguineus* (L.) *Murrill*. It was observed that, among other things, the applied methods of adhesive compound preparation and the applied modifier contents have a significant effect on the properties of the biochemical modified adhesives (in cured state). Moreover the seasoning of the modified adhesives did not have a negative effect on their mechanical properties. In turn, all the quantities tested in the unmodified adhesive decreased after the seasoning. The authors decided to prove the results in the case of using the modified adhesives to preparing adhesive joints.

The aim of this study is to determine the effect of biochemical modification of epoxy adhesive compounds on selected mechanical properties of adhesive joints of hot-dip galvanized steel sheet made with a modified epoxy adhesive. To determine the effect of modification on the mechanical properties of produced adhesive joints, the study also involves running control experiments using an unmodified adhesive as reference. In addition to this, the study also investigates the influence of seasoning on the mechanical properties of adhesive joints prepared using the modified adhesive. The properties of cured adhesives depend on the method of their preparation, the content of modifying agents, and the effect of seasoning on their mechanical properties have been described in a previous work by the authors [45].

## 2. Materials and Methods

### 2.1. Characteristics of Adhesive Joints

The tests were performed on specimens made of hot-dip galvanized steel sheet DX51+Z275–DIN EN 10142 [46] (thickness of zinc coated is 20 μm) and the properties of this steel are listed in Table 1. The zinc coating thicknesses defined according to EN 10346 [47]. Hot-dip galvanizing is particularly durable and effective method of anti-corrosion protection.

**Figure 1:**
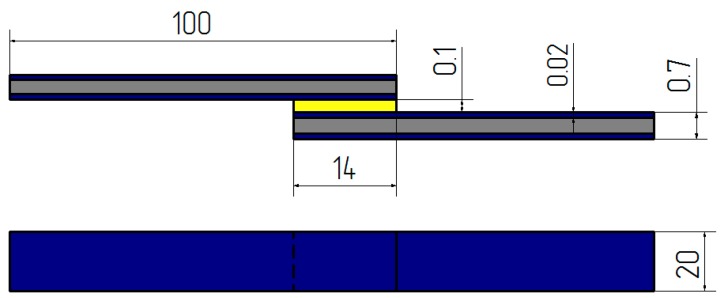
Shape and dimensions of a single-lap adhesive joint of hot-dip galvanized steel sheets.

Hot-dip galvanized steel sheet was used to make 20 × 100 × 0.7 mm specimens which were subjected to adhesive bonding. Strength tests were performed on single-lap adhesive joints, the shape and theoretical dimensions of which are shown in Figure 1.

The length of lap (14 mm) of adhesive joints was determined by calculating the limit lap length (l_gr_) which defines the limit strength of the adhesive joint. The thickness of adhesive layer is 0.1 mm.

### 2.2. Surafce Treatment of Adherends

Given the effect of ambient temperature, air humidity and load on the quality of adhesive joints, it is critical that experiments be run under suitable conditions. Variations in ambient parameters were controlled using a thermometer and hygrometer. Adhesive joints were produced in ambient temperature ranging from 24 to 26 °C, while the humidity ranged from 31 to 32%. They were subjected to a load set to 0.03 MPa.

Adherends were prepared by mechanical treatment with the P320 sandpaper followed by degreasing. Surface roughening by abrasive papers consisted in performing thirty circular movements on each sample. Next, they were degreased with Loctite 7063 to cleanse and degrease the substrates prior to adhesive bonding. The degreasing operation enabled the removal of remnants in the cavities produced due to treatment by abrasive tools. 

The degreasing was performed in several stages:

- the degreasing agent was sprayed in the point for adhesive bonding;
- wet substrates were wiped dry with a clean towel to remove impurities; and
- the above operations were repeated twice, and after the final application of the degreasing agent was left to evaporate (approx. 1 min).

Following the surface preparation process, the adhesive was applied after the degreasing agent dried up. This was followed by performing successive assembly operations, i.e., stabilization of the adherends using a jig [49]. 

### 2.3. Characteristics of Unmodified and Modified Adhesives

The experiments on the adhesive bonding of hot-dip galvanized steel sheet adhesive joints were prepared using an adhesive containing epoxy resin and curing agent in a 1:1 stoichiometric ratio. Epidian 53 epoxy resin and PAC curing agent are commercial products (manufactured by Organika-Sarzyna, Nowa Sarzyna, Poland [50]). The denotation of adhesive is given in Table 2. 

Epidian 53 is a liquid styrene-modified epoxy resin. It has a low viscosity (900–1500 mPa·s at 25 °C), average reactivity, and high insulation properties. It is produced by thinning Epidian 5 (the basis epoxy resin) with styrene in a quantity ranging from 13 ns to 15 ns [50]. The density of this epoxy resin is 1.11–1.15 g/cm^3^ at 20 °C and the number of epoxy is 0.41. The curing of an adhesive compound at an elevated temperature significantly accelerates polyreaction [50]. 

PAC is a modified polyamide curing agent. This curing agent is forming by the polycondensation of polyamine with dimers of unsaturated fatty acid methyl esters. It is primarily used for modifying and curing low molecular weight epoxy resins and compounds based on these epoxy resins. The usage of PAC results in a higher elasticity of cured epoxy compounds and their increased impact strength. In room temperature, the life of an epoxy compound containing this curing agent amounts to several hours, while the total cure time is 4–7 days. To accelerate polyreaction, the curing process can be run at the temperature of about 60 °C for 6–8 h [50]. The detailed characteristics of epoxy resin and the curing agent was presented in [46].

A adhesive compound of the epoxy resin and the PAC curing agent in 1:1 ratio (Epidian 53/PAC/1:1) was modified by a biological material in the form of lyophilized fungal fractions or materials preparation containing low molecular weight secondary metabolites of lignocellulose-degrading white rot fungi (WRF) *P. sanguineus* (L.) *Murrill*. Lyophilized fraction of low molecular weight metabolites (LMS 186) was added in concentrations listed in Table 3.

The preparation of the adhesive with the modifying agent involved the use of two following methods:

- **Method I.** The epoxy resin and the curing agent were mixed with the fungal material in the tested concentration (Table 3). The whole was thoroughly mixed and used for the adhesive bonding of specimens.
- **Method II.** The resin was mixed with mortar-grounded lyophilized post-culture liquid of the tested concentration (Table 3). Following thorough mixing, the poliaminoamide curing agent was added (according to stoichiometric ratio). The whole was then mixed again and used for the adhesive bonding of specimens.

The epoxy adhesive compounds were prepared according to the two methods (listed above) by mechanical mixing with a specially contoured mixer operated at a speed of 460 rev/min. The mixing time was 2 min. After that, degassing was performed. 

### 2.4. Conditions of Production, Seasoning and Investigation of Adhesive Joints

Adhesive joints were produced under the following technological conditions:

- ambient temperature: 24–26 °C;
- relative air humidity: 31–32%;
- load at curing: 0.03 MPa.

The specimens of hot-dip galvanized steel sheet adhesive joints were cured at single stage at the same temperature and humidity for 7 days with Methods I and II (Variants A and B), while with Method I in Variant C adhesive joints were cured for 7 days at the ambient temperature and the seasoned for two months at the temperature of 50 °C and humidity of 50%. 

The conditions of hot-dip galvanized steel sheet adhesive joints are listed in Table 4.

Following the curing and seasoning process (Table 4), the strength tests were performed to determine hot-dip galvanized steel sheet adhesive joints shear strength. The experimental tests in which the single-lap adhesive joints undergo failure were performed using the Zwick/Roell Z150 testing machine in compliance with DIN EN 1465 [51] and at a speed of 5 mm/min. The specimens of made joints were fixed in the screw-wedge clamps of the testing machine. The strength tests were performed for 8 to 12 adhesive joints in 3 test runs per each variant of adhesive joints preparation (epoxy adhesive preparation method and seasoning period) and in 5 test runs taking account of modified and unmodified epoxy adhesives (5 × 10 test runs). The total amount of tested adhesive joints amounted to 150 items. The basic statistics of the results were made. The mean and standard deviation were determined, rejecting the extreme values of the obtained results.

## 3. Results and Discussion

### 3.1. The Effect of Adhesive Preparation Method on Adhesive Joints Strength (Variant A and Variant B)

**Figure 2:**
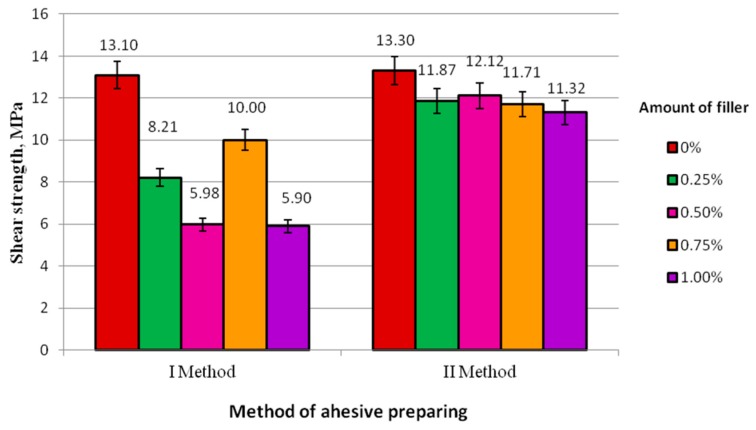
Shear strength of adhesive joints produced according to Methods I and II.

**Figure 3:**
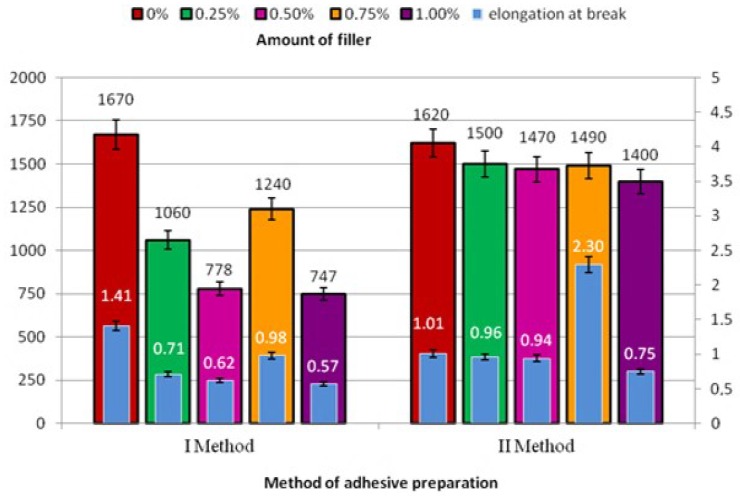
Maximum force and elongation of adhesive joints produced according to Methods I and II.

A comparison of selected mechanical properties (shear strength, maximum force, and elongation) of adhesive joints produced according to Method I (Variant A) and the Method II (Variant B) is given in Figure 2 and Figure 3.

The results obtained for Method I given in Figure 2 reveal that the hot-dip galvanized steel sheet adhesive joints made by the unmodified adhesive have the highest shear strength in all tested cases (13.10 MPa) compared to the adhesive joints made by the modified adhesive. As for adhesive joints produced by the modified adhesive, the highest strength was observed for adhesive joints prepared by the adhesive containing 0.75% of the modifier (10.00 MPa), while the lowest strength, lower by about 40%, was exhibited by adhesive joints produced using the adhesive with 1% and 0.50% of the modifier. The strength of adhesive joints produced using the adhesive containing 0.25% of the modifier is 82% of the strength of adhesive joints produced using the adhesive with 0.75% of the modifier. On investigating Method II of adhesive preparation, it was found that the mean strength of all adhesive joints produced using modified adhesives with the tested modifier concentrations: 0.25% (11.87 MPa); 0.50% (12.12 MPa); 0.75% (11.71 MPa); and 1% (11.32 MPa), is lower than that of the referential specimens. Nonetheless, there are no such significant differences here, as was the case with Method I. The strength of adhesive joints produced with the adhesive containing 0.50% of the modifier is 91% of the strength of the referential adhesive joints. As for the modified adhesives, the highest strength was observed for adhesive joints produced using the adhesive with 0.50% of the modifier. However, the differences in strength of adhesive joints produced using the modified adhesives are not significant. The results of shear strength range from 11.32 MPa to 12.12 MPa.

Comparing both methods of adhesive preparation it was noted that in both cases the strength of adhesive joints made using the modified adhesive with varying modifier contents is lower than that yielded in the control experiments (Method I—13.10 MPa; Method II—13.30 MPa). The strength of adhesive joints produced with adhesives prepared according to Method II is much higher than that of adhesives joints produced according to Method I. The highest difference can be observed comparing the control experiment result (13.10 MPa) of the adhesive joints produced according to Method I with the result of the specimens with additive concentration of 1% (5.90 MPa)—here the difference is 65%. It was also observed that Method II yield higher repeatability of strength results than Method I. It can therefore be claimed that the application of Method II enables producing adhesive joints with much more uniform properties.

As for Method I, in all cases of adhesive joints made using the modified adhesive with the following modifier concentrations: 0.25% (1060 N); 0.50% (778 N); 0.75% (1240 N); and 1.00% (747 N), the obtained values of standard force and elongation are lower than those yielded in the control experiment, i.e., standard force—1670 N and elongation—1.41 mm (Figure 3). The elongation observed for adhesive joints produced according to Method I can indicate reduced elasticity of the adhesive due to the addition of lyophilized fungal material. The highest elongation was observed for adhesive joints produced using adhesive containing 0.75% of the modifier (0.98 mm), which is 70% of elongation of the adhesive joints produced using the unmodified adhesive. The values of elongation of the adhesive joint specimens were slightly scattered from 0.57 mm to 0.98 mm.

Regarding Method II, it was observed that the applied method of adhesive mixing resulted in forces which do not significantly vary, as was the case with Method I. The highest force is observed for the referential adhesive joints (1620 N); however, values of this force are similar in three cases of adhesive joints produced with the modified adhesive, i.e., 0.25% (1500 N); 0.50% (1470 N); and 0.75% (1490 N), which is 93%, 91%, and 92% of the maximum force of the referential specimens, respectively. The application of Method II also led to higher elongation for every applied modifier concentration compared to that of the specimens of adhesive joints produced according to Method II. The highest adhesive joint elongation was obtained for the specimens produced by the modified adhesive with a concentration of 0.75% of the modifying agent (2.30 mm). Although in other cases the elongation of the adhesive joints prepared by modified adhesives is smaller than that of the specimens in the control experiment, the difference is not significant.

The chart in Figure 3 shows that the maximum force of adhesive joint specimens produced using adhesives with varying concentrations according to Methods I and II are lower than those of the control experiments (Method I—1670 N and Method II—1620 N). The highest difference can be observed in the results of specimens modified by Method I. The standard force for the concentrations 0.50% and 1% is approximately two times smaller than that of the referential specimen. The elongation of the zero specimen (1.41 mm) regarding Method I is higher than that for the applied concentrations, ranging between 0.43 mm and 0.84 mm. With Method II, mean elongation of the specimens with a concentration of 0.75% (2.30 mm) is two times higher than that of the 0% specimens (1.01 mm), and the values of elongation of adhesive joints produced using the modified adhesive with varying concentrations of the modifying agent do not reveal any significant difference compared to the results of specimens in the control experiments. The data comparison (Figure 3) reveals that the highest elongation can be observed for the specimens produced by Method II for a concentration of 0.75% (2.30 mm).

### 3.2. Failure Patterns of Adhesive Joints

**Figure 4:**
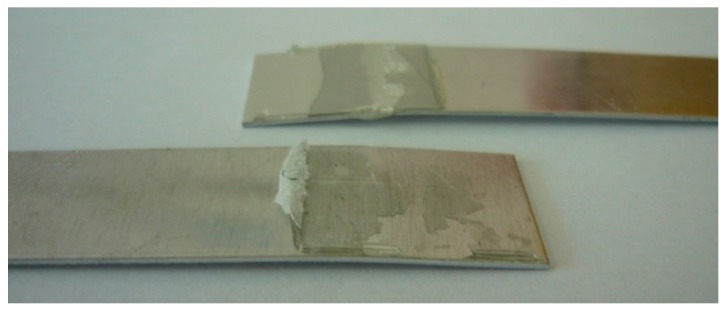
View of adhesive joints after strength test—the view of SCF—special cohesion failure.

The assessment of adhesive joint failure was conducted in compliance with the EN ISO 10365 standard [52]. It was found that the most of tested specimens reveal the presence of SCF failure (Table 5 and Table 6 and Figure 4).

It can be observed that the addition of the modifying agent (lyophilized fungal material) does not affect the failure pattern of aluminum adhesive joints of alloy steel sheets compared to joints produced by the unmodified adhesive. Therefore, the preparation method of the adhesive cured for 7 days in ambient conditions does not affect failure pattern.

### 3.3. Effect of Seasoning on Adhesive Joints Strength (Variant A and Variant C)

**Figure 5:**
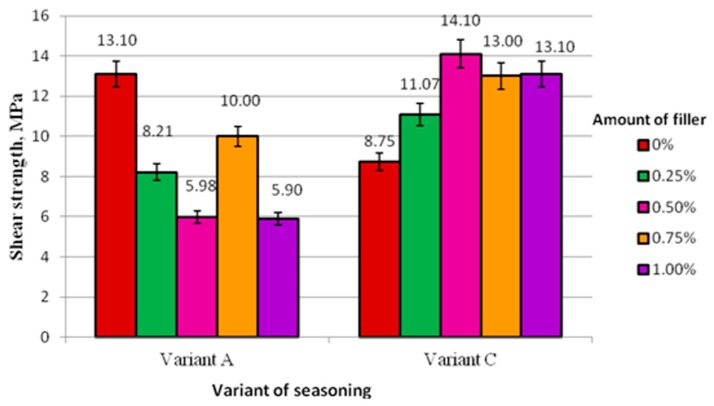
Tensile strength of adhesive joints produced according to Methods I and including Variant A and Variant C seasoning.

A comparison of adhesive joint shear strength for specimens produced according to Method I subjected to curing at the ambient temperature (Variant A) and subjected to curing at the ambient temperature and the seasoning the elevated temperature (Variant C) is shown in Figure 5.

The chart in Figure 5 reveals that the specimens produced by Method I and subjected to seasoning (Variant C) have a higher shear strength compared to control specimens (8.75 MPa). The strength of adhesive joints produced with modified adhesives exceeds the strength of referential specimens as follows for respective concentrations: 0.25–27%; 0.50–61%; 0.75–49%; and 1–50%. These results clearly differ from the results of specimens produced using Method I (Variant A), where the specimens prepared using unmodified (referential) adhesive have the highest strength.

Examining the method I of preparation of modified adhesive, it was observed that seasoning (Variant C) has a definitely positive effect on the strength of adhesive joints of hot-dip galvanized steel sheet sheets. In each case of modified adhesive with different modifier contents, it can be observed that the strength of the specimens after seasoning is higher under applied conditions. This increase is as follows for respective concentrations: 0.25% for 26%; 0.50% for 58%; 0.75% for 23%; and 1% for 55%. As for adhesive joints made with unmodified adhesive, their strength after seasoning is 67% of the strength of adhesive joints that were not subjected to seasoning (13.10 MPa).

**Figure 6:**
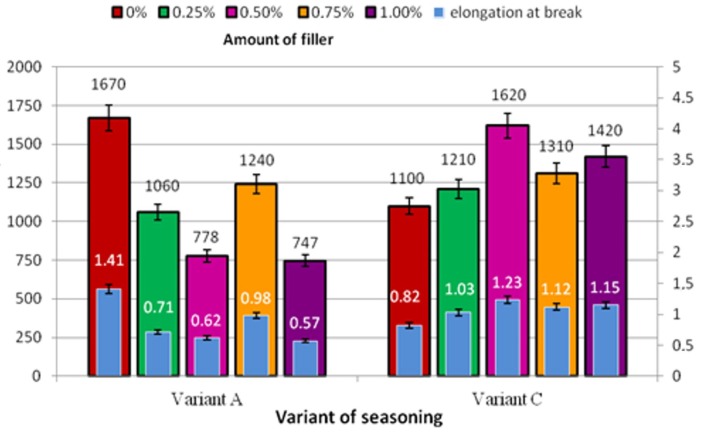
Maximum force and elongation at break of adhesive joints produced according to Methods I and including Variant A and Variant C seasoning.

Variations in the failure force of adhesive joints subjected to seasoning in the climatic chamber, produced according to Method I (Variant A and C) using adhesive with varying concentrations of biological material are shown in a chart in Figure 6.

For all applied concentrations: 0.25% (1210 N); 0.50% (1620 N); 0.75% (1310 N); and 1% (1420 N), the values of standard force and elongation are higher than the results obtained in the control experiment, in which the standard force is 1100 N and the elongation is 0.82 mm. The values of elongation reveal a small scatter (Figure 6). The values of elongation of the adhesive joints specimens produced according to Variant C are higher than the results of both the control experiment and those obtained for the specimens produced by Method I in Variant A, where (Figure 6, Method I) elongation is smaller for each applied concentration than in the blank test (0%). The elongation of modified adhesive-produced adhesive joints subjected to seasoning is higher than the elongation of adhesive joints produced by the unmodified adhesive, for respective concentrations of: 0.25–20%; 0.50–37%; 0.75–27%; and 1–29%. The results of adhesive joint specimens produced with modified adhesives according to Variants A and C reveal the following differences for respective concentrations: 0.25–31%; 0.50–49%; 0.75–12.5%; and 1–50%. Moreover, it was observed that the elongation of adhesive joints produced using the unmodified adhesive and subjected to seasoning (0.82 mm) amounts to 58% of elongation of the adhesive joints that were not subjected to seasoning (1.41 mm).

Based on the results it can be concluded that seasoning had a positive effect on the mechanical properties of hot-dip galvanized steel sheet adhesive joints produced by the modified adhesives for all applied concentrations of the modifying agent (lyophilized fungal material). Seasoning had a negative effect on the strength of adhesive joints produced using the unmodified adhesive. Based on the results of previous tests [46], it can be supposed that the use of metabolites derived from fungal cultures as modifiers for epoxy compounds could have a positive effect on degradation processes of modified adhesive compounds. It can be claimed that the high antioxidative potential of fungal modifiers and their qualitative composition (the content of phenolic compounds and low molecular weight of active proteins or carbohydrates) can notably change the properties of the modified epoxy adhesive compounds used to make adhesive joints. 

## 4. Conclusions

The results of strength tests of specimens of adhesive joints performed on the testing machine reveal that the type of method for adhesive compound modification has a significant effect on some mechanical properties of adhesive joints. On comparing the results it was possible to determine which of the applied methods leads to decreased properties of the applied adhesive compound. Adhesive joint strength is also connected with elongation of the adhesive layer which occurred during the tests due to the impact of failure force.

■ The strength of adhesive joints decreases with increasing the modifier’s concentration compared to the results of the control experiment.
■ The second method of preparing the epoxy adhesive compounds (Method II), when in the first step the resin was mixed with mortar-grounded lyophilized post-culture liquid and after that with poliaminoamide curing agent, allowing us to obtain the higher strength of tested adhesive joints.
■ The results of the effect of seasoning on adhesive joint strength demonstrate that seasoning has a positive impact on the mechanical properties of adhesive joints made of hot-dip galvanized steel sheets using modified adhesives; this observation is true for all applied concentrations of the modifying agent, i.e., lyophilized fungal material. In contrast, seasoning has a negative effect on the strength of adhesive joints produced with the unmodified adhesive.
■ As a result, the strength of adhesive joints produced using the modified adhesive is not affected by elevated temperature or humidity. The modified specimens put in the climatic chamber exhibit higher strength and elongation with increasing the concentration of the modifying agent, compared to the results of control experiments.

Summing up, the quality of produced adhesive joints is affected by both the applied method for modification of the adhesive compound and the exposure of specimens to seasoning in the climatic chamber. The experimental results demonstrate that the addition of the biological modifier can lead to reduced adhesive joint strength in ambient conditions, yet at elevated temperature and higher humidity it results in a significant increase in adhesive joint strength. 

The obtained test results also have a practical dimension. The modified adhesive modification allows to achieve both industrial, economic, and ecological benefits. Extending the service life of adhesive joints allows for their appropriate design in a given operating environment. The economic dimension will be manifested in the lower consumption of adhesives (extending the life time—lower costs of adhesive materials), and the ecological dimension is the introduction of natural, non-toxic, and environmentally friendly bioproducts and the production of biodegradable materials will contribute to greater protection of the natural environment.

## Figures and Tables

**Table 1:** Mechanical properties of adherends [48].

Properties	Value
Tensile strength Rm min, MPa	270
Tensile strength Rm max, MPa	500
Elongation min A_80mm_, %	22

**Table 2:** Composition of the unmodified epoxy adhesive.

Epoxy Resin	Curing Agent	Stoichiometric Ratio	Denotation
Epidian 53	PAC	100:100	Epidian 53/PAC/1:1

**Table 3:** Modified epoxy adhesive compounds [46].

Components of Epoxy Adhesive Compound	Control Test (g)	Test Run 1 (g)	Test Run 2 (g)	Test Run 3 (g)	Test Run 4 (g)
Epidian 53	50	50	50	50	50
PAC	50	50	50	50	50
Lyophilized preparation of low molecular weight secondary metabolites	0.00	0.25	0.50	0.75	1.00

**Table 4:** Conditions of hot-dip galvanized steel sheet adhesive joints preparation for the shear strength testing.

Variant	Method of Adhesive Compounds Preparation	Curing Period	Seasoning Period	Seasoning Conditions
Variant A	Method I	7 days	-	Temperature: 23 °C ± 2 °C Humidity: 23 ± 2%
Variant B	Method II		-	
Variant C	Method I		2 months	Temperature: 50 °C ± 1 °C Humidity: 50 ± 1%

**Table 5:** Failure patterns evaluation according to EN ISO 10365 Standard—method I.

Amount of Filler	Adherend			Adhesive			
	SF	CFS	(p) DF	CF	SCF	AF	ACF (p)
	Number of Samples						
0.00%					7	3	
0.25%				2	8		
0.50%				3	7		
0.75%				3	7		
1.00%				1	9		

**Table 6:** Failure patterns evaluation according to EN ISO 10365 Standard—method II.

Amount of Filler	Adherend			Adhesive			
	SF	CFS	(p) DF	CF	SCF	AF	ACF (p)
	Number of Samples						
0.00%				1	5	4	
0.25%				3	7		
0.50%				4	6		
0.75%				3	7		
1.00%				1	9		

Where: SF—Substrate failure, CSF—cohesive substrate failure, (p) DF—delamination failure, CF—cohesion failure, SCF—special cohesion failure, AF—adhesion failure, ACF (p)—adhesion and cohesion failure with peel.
